# Acute Cerebrovascular Accident, Renal Failure, and Thrombotic Microangiopathy in a 27‐Year‐Old Male With Malignant Hypertension

**DOI:** 10.1002/ccr3.70565

**Published:** 2025-06-16

**Authors:** Tatiana Gusan, Angelina Hong, Sarah Kaufman, Luis Santiago, Nathan Zaher

**Affiliations:** ^1^ Department of Internal Medicine HCA Florida Westside Hospital Plantation Florida USA

## Abstract

Hypertensive emergency is an acute, significant elevation of blood pressure accompanied by end‐organ damage. We present a case of a 27‐year‐old gentleman who acquired multiple complications of hypertensive emergency, including renal failure and microangiopathy. This highlights the diverse complications of hypertensive emergency and reviews guideline‐based recommendations for management.

## 1 Introduction

Hypertensive emergency is an acute, significant elevation of blood pressure that is accompanied by signs and symptoms of end organ damage [1]. Hypertensive emergency, along with hypertensive urgency, is part of a clinical syndrome known as hypertensive crises, which normally is the result of inadequately treated or untreated hypertension [1]. The most common cutoffs proposed for hypertensive urgency are systolic blood pressure greater than 180 mmHg or diastolic blood pressure greater than 110 mmHg [2]. Typically, autoregulation allows for fluctuations in blood pressure to maintain a stable blood flow to the brain, heart, and kidneys, but this mechanism fails in hypertensive emergency [3]. The most common clinical manifestations of hypertensive emergency include cardiac ischemia, pulmonary edema, neurologic deficits, aortic dissection, eclampsia in pregnant patients, and acute renal failure [4]. The existing literature, including a 2014 retrospective study by Salkic et al. suggests that the most common symptom in patients presenting with hypertensive crisis is headache, followed by dizziness, and then nausea and vomiting [5].

Rarely, hypertensive emergency can also lead to thrombotic microangiopathy (TMA). Uncontrolled hypertension can induce TMA via endothelial injury and thrombosis of the microvasculature [6]. This thrombosis in the microvasculature typically causes mechanical hemolytic anemia, platelet consumption causing thrombocytopenia, and ischemic organ damage, most commonly associated with renal damage [6]. TMA is associated with renal dysfunction, and hypertensive emergency can produce both chronic nephrosclerosis as well as acute disruptive vascular and glomerular injury with prominent fibrinoid necrosis and thrombosis [5]. The following case describes a young patient who acquired multiple complications of uncontrolled hypertension, including acute renal failure and microangiopathic hemolytic anemia.

## 2 Case History and Examination

The patient is a 27‐year‐old African American gentleman with no known past medical history who presented with complaints of chest pain, bilateral blurry vision, headache, and lower extremity weakness for the past 2 days. He described the chest pain as non‐radiating, substernal, and without any obvious aggravating or alleviating factors, but it improved by the time he arrived at the emergency department. He said the headache was diffuse and episodic. The patient endorsed intermittent periods of blurry vision, but upon arrival reported he was not currently experiencing vision loss symptoms. He took no medications at home. He had no surgical history. He denied alcohol, tobacco, or illicit drug use. He had a family history of hypertension. He had not been following up with a primary care physician.

On presentation, his blood pressure was 266/169 mmHg, heart rate was 99 beats per minute, respiratory rate 16 breaths per minute, and he was afebrile. His BMI was 30.5. On exam, he had normal heart sounds with no ectopy or murmurs appreciated. Lungs were clear to auscultation bilaterally. Abdomen was soft and nontender. He was able to move all extremities and had no focal neurologic deficits. Visual acuity was symmetrical, and visual fields were intact. He had symmetric peripheral pulses on both upper and lower extremities. He was somnolent but oriented to person, place, and time, and was able to follow commands.

Initial bloodwork was significant for a creatinine level of 6.3 mg/dL, blood urea nitrogen of 50 mg/dL, troponin I of 0.466 ng/mL, hemoglobin of 9.3 g/dL, and platelet count of 92,000/uL. His electrocardiogram showed no acute ischemic changes.

## 3 Differential Diagnosis

This patient initially had a very broad differential diagnosis given his multiple complaints in the setting of uncontrolled hypertension with acute kidney injury, anemia, and thrombocytopenia. Initially, there was suspicion for posterior reversible encephalopathy syndrome (PRES) given his new onset vision loss with hypertensive crisis, versus an acute ischemic or hemorrhagic cerebrovascular accident (CVA), although the patient had no appreciable focal deficits on exam [5].

Uncontrolled hypertension can have various etiologies. Primary (essential) hypertension is multifactorial without one clear cause, whereas secondary hypertension can be drug‐induced or related to endocrinologic disorders, chronic kidney disease, severe sleep apnea, among other causes [7]. This patient denied taking any prescription or over‐the‐counter medications like nonsteroidal anti‐inflammatory drugs at home. He was obese as demonstrated by his BMI, but he did not report any significant symptoms consistent with sleep apnea or a history of sleep apnea diagnosis. He did not have a history of endocrinologic diseases that could cause hypertensive crisis, such as Cushing disease (which is screened for utilizing cortisol level testing), pheochromocytoma (diagnosed by checking metanephrine levels), primary hyperaldosteronism (screened for by measuring serum renin and aldosterone levels), or hyperthyroidism (requiring thyroid function testing for diagnosis); however, this required further workup, as he had never undergone diagnostic testing for these conditions [7].

Additionally, his thrombocytopenia and anemia on arrival, in addition to the abnormalities discussed above, raised concerns for severe hematologic diseases such as hemolytic uremic syndrome (HUS) or thrombotic thrombocytopenic purpura (TTP) [6]. However, infectious causes of HUS typically occur in patients with complaints of bloody diarrhea, nausea, and/or vomiting who have a recent history of travel or ingestion of poorly cooked beef [6]. Patients with TTP are critically ill and classically (but not always) also develop fevers, which our patient did not. Atypical HUS was also considered in the differential diagnoses, as it encompasses the TMAs that are not TTP or the typical HUS that is caused by Shigella toxin from *Escherichia coli* infection [8]. Primary atypical HUS is caused by dysregulation of the complement cascade, and diagnosis can be confirmed with genetic testing or by measuring complement variants or autoantibodies, although abnormalities in complement assays are not always seen uniformly in patients with atypical HUS [8]. The differential diagnosis also included other causes of thrombotic microangiopathy such as hypertensive emergency, autoimmune phenomena, or drug‐induced processes.

Although the patient was only 27 years old with chest pain, the differential diagnosis also included an acute myocardial infarction as his initial labs showed a moderately elevated troponin level. However, his chest pain improved by the time he arrived, was not worsened by exertion, and his troponin elevation appeared to be multifactorial due to his other comorbidities, including acute renal failure. Aortic dissection was also considered as a cause of his chest pain with uncontrolled blood pressure. This was less likely as his chest pain was improved on arrival, not severe or tearing in quality, did not radiate to his back, and he had symmetric pulses and blood pressure readings on right and left extremities.

## 4 Conclusion and Results

**FIGURE 1:**
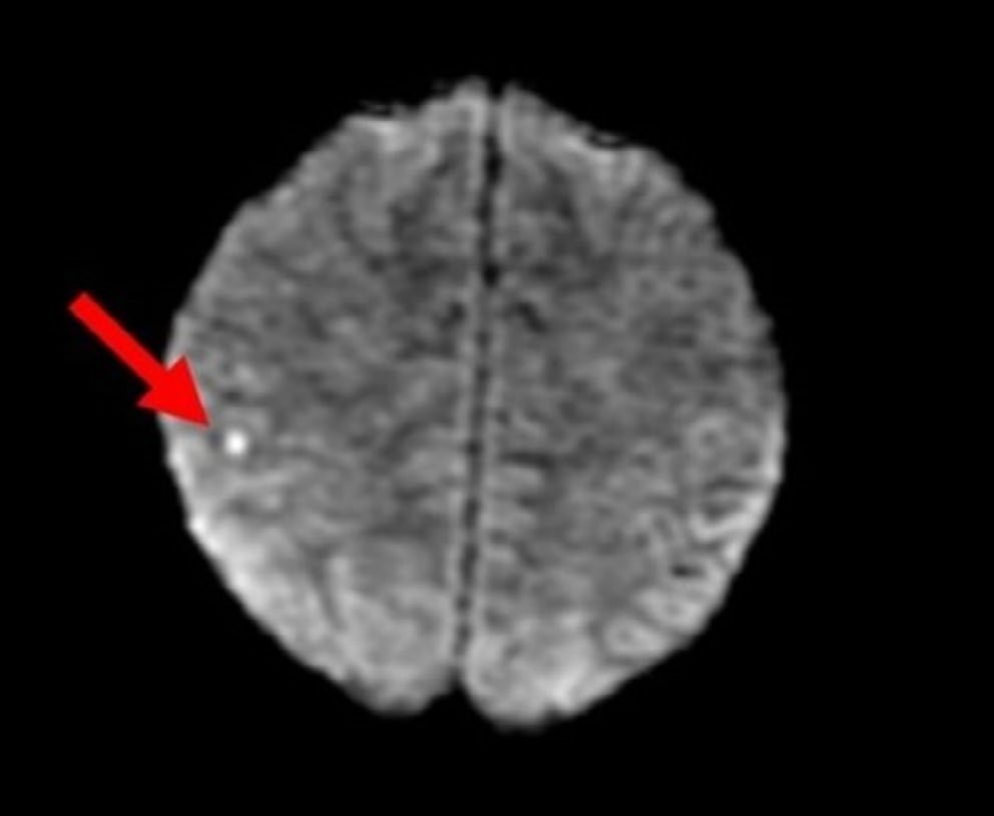
Axial magnetic resonance imaging of the brain showing a 3 mm punctate acute ischemic infarct in the right parietal lobe (arrow).

**FIGURE 2:**
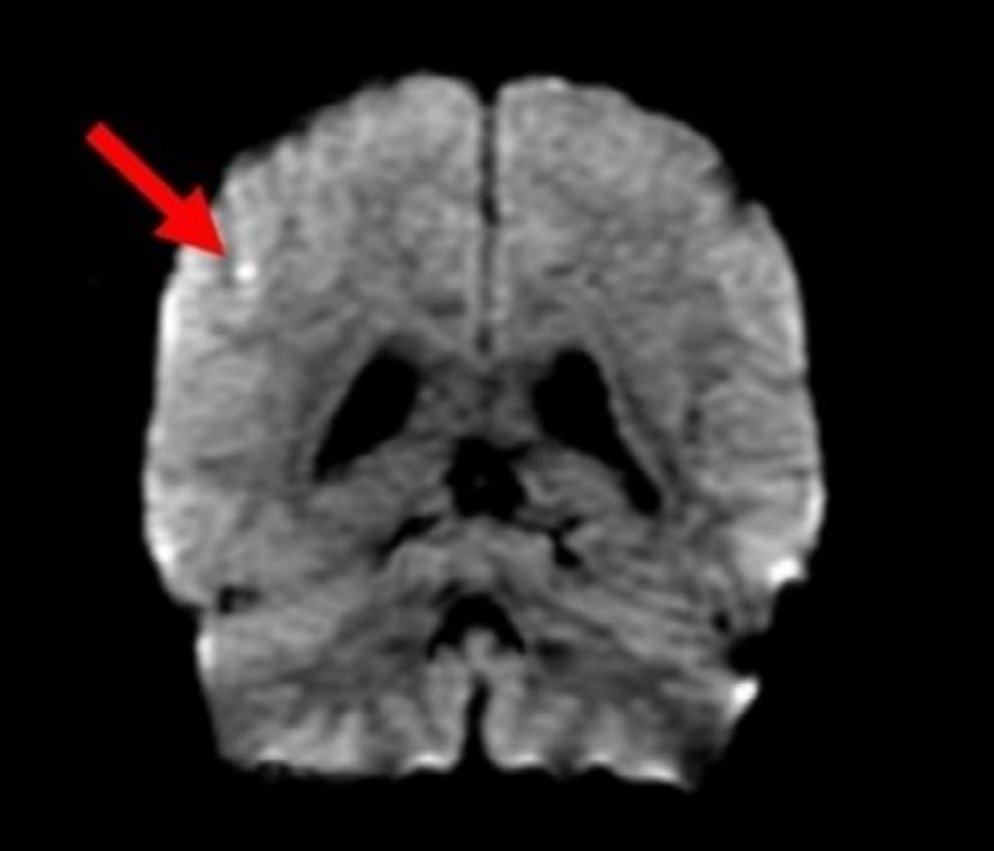
Coronal magnetic resonance imaging of the brain also demonstrating the 3 mm punctate acute ischemic infarct in the right parietal lobe (arrow).

The patient underwent extensive laboratory and imaging testing for further investigation of the broad differential discussed above. Chest X‐ray showed no effusions or infiltrates, and there was no widened mediastinum. His computed tomography of the brain without contrast showed no hemorrhage. However, magnetic resonance imaging of the brain showed a 3 mm punctate acute ischemic infarct in the right parietal lobe (Figures 1 and 2). He subsequently had a transthoracic echocardiogram with normal results, followed by a transesophageal echocardiogram that was also unimpressive. His renal ultrasound showed no evidence of renal artery stenosis. The consideration of a renal biopsy was discussed with the patient for further evaluation of his acute kidney injury, but the patient declined the procedure during hospitalization.

On further laboratory studies, his hemolysis workup revealed low haptoglobin, elevated lactate dehydrogenase levels, and elevated total bilirubin that was predominantly indirect bilirubin. Schistocytes were noted on the peripheral blood smear. Urine drug screen was negative. ADAMTS13 level was within normal limits His autoimmune workup was negative. His thyroid stimulating hormone and free T4 levels were within normal limits. Serum renin, aldosterone, morning cortisol, and free metanephrines were also normal. HIV and hepatitis screening were negative. CH50, C3, and C4 levels were in the normal range.

He was started on a nicardipine drip for blood pressure control, then he was transitioned to oral antihypertensives the following day. Renal function gradually improved and he did not require renal replacement therapy during his hospital course. His troponin levels downtrended and chest pain resolved fully. He was discharged on a baby aspirin, a statin, and a blood pressure regimen comprised of metoprolol tartrate, hydralazine, and nifedipine. Given his severe acute kidney injury, he was advised to follow up outpatient for repeat labs and evaluation for modifying his antihypertensive regimen with other medications such as diuretics and angiotensin‐converting enzyme (ACE) inhibitors or angiotensin receptor blockers (ARB), which can potentially worsen acute kidney injury [4].

His symptoms did not recur, and he remained adherent to his medications. His bloodwork 3 months later showed an improvement in creatinine from 6.3 mg/dL from when he was hospitalized to 2.4 mg/dL. His hemoglobin improved from 9.3 g/dL to 11.4 g/dL.

## 5 Discussion

This is a case of uncontrolled hypertension in a young African American patient with multiple manifestations of end organ damage. Hypertension, now defined as greater than 120/80 mmHg by the 2017 American College of Cardiology and American Heart Association Task Force on Clinical Practice, is estimated to be prevalent in 46% of the adult population [3]. It is estimated that 3% of all emergency department visits are due to hypertensive urgency, and hypertensive emergency occurs in about 1% of the hypertensive population [6, 9]. The average age of patients who are admitted to the hospital due to hypertensive emergency is 55–60 years old [3]. The incidence of hypertensive emergency is higher in African Americans and Asians [3, 9]. Males are twice as likely to be affected as females [9].

Our case shows the rare presentation of thrombotic microangiopathy from hypertensive emergency. The various etiologies of thrombotic microangiopathy include thrombotic thrombocytopenic purpura (TTP), Shiga toxin‐mediated hemolytic uremic syndrome, complement‐mediated TMA (C‐TMA or also called primary atypical HUS), and drug‐induced TMA. Systemic disorders that can cause thrombotic microangiopathy, other than hypertensive emergency, are systemic lupus erythematosus, pre‐eclampsia with severe features, hemolysis, elevated liver enzymes, and low platelet count (HELLP) syndrome, or complications following hematopoietic stem cell or solid organ transplantation [6, 9, 10]. Khanal et al.'s 2015 literature review examined 19 cases of hypertension induced thrombotic microangiopathy. Their study found that the median age of diagnosis was 38‐year‐old, all of the patients had significant renal dysfunction, with 54% requiring hemodialysis, and moderate thrombocytopenia. 58% of the cases had a history of hypertension [11]. Thus, the patient discussed in this case report is unique in that his age was significantly younger than the typical age range in which hypertensive emergency with TMA presents.

If the patient is known to have acute worsening of organ function, then the blood pressure needs to be decreased aggressively. In all other cases, the blood pressure should be lowered gradually to avoid dysfunction of the brain from low perfusion pressure. During the first hour, blood pressure should be reduced by 10%–15% [12, 13]. Within the first 2–3 h, the treatment should decrease the blood pressure by 25% to prevent cerebral hypoperfusion [12, 13]. The reduction must be done gradually because there is a threshold of hypoperfusion in the body's autoregulative ability that is approximately 20%–25% below the existing blood pressure [12, 13]. If the patient has an aortic dissection, however, the systolic blood pressure should be < 120 mmHg within the first 20 min of treatment [11]. The intravenous antihypertensives can be transitioned to oral antihypertensives, typically within the first 6–12 h of treatment [12]. In contrast, in hypertensive urgency, without signs of acute organ damage, the blood pressure can be lowered within 24–48 h [14]. The ideal oral antihypertensive regimen for these patients is customized based on the etiology of the hypertension and the individual's risk factors and comorbidities. In general, patients with resistant hypertension benefit from combination therapy with a renin‐angiotensin‐aldosterone system (RAAS) inhibitor such as an ACE inhibitor or ARB, calcium channel blocker, and a diuretic, but there is also evidence that beta‐blockers and vasodilators (especially in African American patients with chronic kidney disease) such as hydralazine can be very effective agents for blood pressure control [7]. This patient was started on a RAAS inhibitor in the outpatient setting once his renal function improved, as this is beneficial therapy for patients with comorbidities such as chronic kidney disease and malignant hypertension [7, 8].

The first‐line treatment of TMA varies depending on the cause. Plasma exchange is the standard therapy for TMA secondary to TTP [15]. However, when it is caused by hypertensive emergency, it can be treated with antihypertensives alone and plasma exchange is generally not needed [15]. It is suggested that plasma exchange should be done if the platelet count is less than 50,000 cells/μL and there is high suspicion for TTP, even if the ADAMTS13 level (which, if found to have decreased levels, confirms the diagnosis of TTP) has not yet resulted [16]. In contrast to the management of TTP, standard treatment for primary atypical/complement‐mediated HUS is eculizumab, a monoclonal antibody targeting complement C5 [6]. Recent literature now suggests that there is a significant overlap between patients with malignant hypertension and TMA and those with primary atypical HUS; Cavero et al.'s 2019 study found that 36 of 55 patients with primary atypical HUS presented with grade 2 or 3 hypertension [8]. Research is ongoing about this complex condition, but studies thus far suggest that complement dysregulation may be more common in patients with hypertension‐associated TMA than previously thought, and thus these patients could also benefit from eculizumab therapy [8]. Timmermans et al.'s 2017 study demonstrated that nine patients with hypertension‐associated TMA were found to share pathogenic mutations in complement genes [17]. African Americans may have a higher likelihood of developing TMA caused by severe hypertension [8]. The patient in our case had normal serum CH50, C3 and C4 complement levels, but he did not undergo genetic testing during his hospitalization to further evaluate for complement abnormalities. However, given his rapid improvement both clinically and on bloodwork (with stabilization of renal function, platelet count and hemoglobin), with antihypertensive management alone, he did not receive eculizumab therapy.

In summary, this is an uncommon case of hypertensive emergency in a 27‐year‐old male, with no known history of hypertension and no illicit drug use, resulting in acute kidney failure, cerebrovascular accident, and thrombotic microangiopathy. Our patient's case was particularly unique given his young age of presentation with hypertensive emergency and TMA, and no known history of prior hypertension.

## Author Contributions

**Tatiana Gusan:** conceptualization, writing – original draft, writing – review and editing. **Angelina Hong:** writing – original draft, writing – review and editing. **Sarah Kaufman:** writing – original draft, writing – review and editing. **Luis Santiago:** resources, writing – review and editing. **Nathan Zaher:** supervision, writing – review and editing.

## Consent

We obtained written informed consent from the patient for the publication of this case. The patient himself signed the consent form with his full legal name.

## Conflicts of Interest

The authors declare no conflicts of interest.

## Data Availability

Data sharing is not applicable to this article, as no datasets were generated or analyzed during the current study.
